# Stage 2 Registered Report: Variation in neurodevelopmental outcomes in children with sex chromosome trisomies: testing the double hit hypothesis

**DOI:** 10.12688/wellcomeopenres.14677.4

**Published:** 2021-06-01

**Authors:** Dianne F. Newbury, Nuala H. Simpson, Paul A. Thompson, Dorothy V. M. Bishop

**Affiliations:** 1 Department of Biological and Medical Sciences, Oxford Brookes University, Oxford, OX3 0BP, UK; 2 Department of Experimental Psychology, University of Oxford, Oxford, OX2 6GG, UK

**Keywords:** sex chromosome trisomy, language, autism spectrum disorder, neuroligin, synapse, X chromosome, Y chromosome

## Abstract

**Background**: The presence of an extra sex chromosome is associated with an increased rate of neurodevelopmental difficulties involving language. The 'double hit' hypothesis proposes that the adverse impact of the extra sex chromosome is amplified when genes that are expressed from the sex chromosomes interact with autosomal variants that usually have only mild effects. We predicted that the impact of an additional sex chromosome on neurodevelopment would depend on common autosomal variants involved in synaptic functions.

**Methods:** We analysed data from 130 children with sex chromosome trisomies (SCTs: 42 girls with trisomy X, 43 boys with Klinefelter syndrome, and 45 boys with XYY). Two comparison groups were formed from 370 children from a twin study. Three indicators of phenotype were: (i) Standard score on a test of nonword repetition; (ii). A language factor score derived from a test battery; (iii) A general scale of neurodevelopmental challenges based on all available information. Preselected regions of two genes, *CNTNAP2* and *NRXN1*, were tested for association with neurodevelopmental outcomes using Generalised Structural Component Analysis.

**Results:** There was wide phenotypic variation in the SCT group, as well as overall impairment on all three phenotypic measures. There was no association of phenotype with *CNTNAP2* or *NRXN1 *variants in either the SCT group or the comparison groups. Supplementary analyses found no indication of any impact of trisomy type on the results, and exploratory analyses of individual SNPs confirmed the lack of association.

**Conclusions: **We cannot rule out that a double hit may be implicated in the phenotypic variability in children with SCTs, but our analysis does not find any support for the idea that common variants in *CNTNAP2* or *NRXN1 *are associated with the severity of language and neurodevelopmental impairments that often accompany an extra X or Y chromosome.

**Stage 1 report:** http://dx.doi.org/10.12688/wellcomeopenres.13828.2

## Introduction

Developmental language disorder (DLD), a condition in which there are unexplained and persistent difficulties with language acquisition, affects around 7% of children ( Norbury *et al*., 2016). Family studies show that DLD runs in families ( Bishop, 2008), yet it has proved hard to identify any genetic or environmental factors that substantially increase risk. One reason is that DLD appears to be a complex multifactorial disorder where influences of individual genetic variants (alleles) are typically of small effect and may interact with other genetic factors and with the environment. Indeed, the ways in which disorders pattern in families suggest that common genetic variants that confer risk of language disorder may lead to an autistic phenotype when they occur with other genetic risk factors ( Bishop, 2010). Thus the specific phenotype can depend on the constellation of genetic variants, rather than there being separate risk factors for DLD and autism spectrum disorder (ASD).

Rather than recruiting increasingly large numbers to try to find reliable associations between language disorders and genetic variants in genome-wide studies, one way forward is to study rare disorders that have a large impact on the phenotype, which may point to functional pathways involved in more common forms of disorder. One instance of a striking association between a genetic condition and language disorder in children of normal intelligence is provided by the sex chromosome trisomies (SCTs), each of which affects 1–1.5 per 1000 children ( Nielsen & Wohlert, 1991). In the 1960s, research was initiated to investigate neurodevelopmental outcomes of children with SCT detected on neonatal screening. A systematic review of these studies showed that in all three trisomies there were high rates of speech and language impairment, motor problems, and educational difficulties, despite IQ being within normal limits in most cases ( Leggett *et al*., 2010). Furthermore, studies of samples who have developmental language disorder of unknown cause find an increased prevalence of sex chromosome trisomies ( Simpson *et al*., 2014).

In a study of children with sex chromosome trisomies identified on prenatal screening, Bishop *et al*. (2011) found that 7 of 30 (24%) girls with karyotype 47,XXX, 9 of 19 (47%) boys with 47, XXY and 15 of 21 (71%) boys with 47,XYY had a history of speech and language-therapy, compared with rates of 4% in sisters and 18% in brothers. Furthermore, this same study found that 2 of 19 (11%) boys with 47,XXY, and 4 of 21 (20%) boys with 47,XYY had received a diagnosis of ASD, compared with an estimated national prevalence rate of 0.2% in girls and 0.6% in boys. In addition, many children with SCTs who were not diagnosed with ASD had evidence of communication difficulties on parental report, including pragmatic (autistic-like) problems, in all three karyotypes. More recent research has provided further evidence of a link with autism as well as other neurodevelopmental disorders in boys with a sex chromosome trisomy ( Ross *et al*., 2012).

The impact of a trisomy is influenced by distinctive characteristics of the sex chromosomes. In most cases, the phenotypic effects of SCTs are much less severe than the impact of an autosomal trisomy: Down syndrome (trisomy 21) usually leads to intellectual disability, and most other trisomies are lethal. Viable trisomies usually involve small chromosomes with a low gene count (for example the Y chromosome), where the effects associated with altered gene dosage are less severe. An exception to this rule is the X chromosome. The X chromosome has a relatively high gene count, but the impact of a duplication is relatively mild because mechanisms of inactivation have evolved, such that in typical females, only one copy is active, and in effect, both males and females have one set of functional genes from this chromosome. In trisomies that involve the X chromosome, two copies are inactivated, largely negating the presence of additional genetic material. There are, however, exceptions to this rule, with between 12–20% genes escaping inactivation to some extent: These include genes in the pseudo-autosomal region, and other genes that have homologues on the Y chromosome ( Carrel & Brown, 2017).

The fact that there is an increase in problems affecting speech, language and communication in all three sex chromosome trisomies suggests there is an adverse impact of an additional copy of a gene that is expressed and has homologous forms on the X and Y chromosomes. *Neuroligin-4 (NLGN4)* is a strong candidate for such a gene, for several reasons ( Bishop *et al*., 2011). First, *NLGN4X*, located on Xp22, at least partly escapes inactivation ( Berletch *et al*., 2011). Second there is a homologous gene, *NLGN4Y* on the Y chromosome at Yq11.2. Third, *neuroligins* are expressed in brain, as well as other tested tissues ( Abrahams & Geschwind, 2010; Jamain *et al*., 2003). Fourth, as reviewed by Cao & Tabuchi (2017), mutations of *NLGN4* have been linked to ASD ( Jamain *et al*., 2003; Laumonnier *et al*., 2004; Lawson-Yuen *et al*., 2008; Marshall *et al*., 2008; Pampanos *et al*., 2009; Talebizadeh *et al*., 2006; Yan *et al*., 2008) – although this finding is inconsistent and other studies have not found autism in those with mutations of *NLGN4* ( Chocholska *et al*., 2006; Macarov *et al*., 2007), or have failed to find abnormalities of *NLGN4* in those with autism ( Blasi *et al*., 2006; Gauthier *et al*., 2005; Liu *et al*., 2013; Vincent *et al*., 2004; Yanagi *et al*., 2012). Fifth, neuroligins are postsynaptic transmembrane proteins that mediate development of functional synapses between neurons and are in the same functional network as neurexins ( Craig & Kang, 2007), which have also been implicated in both DLD and ASD ( Vernes *et al*., 2008). Jamain *et al*. (2003) proposed that a defect in *NLGN4* may abolish formation or function of synapses involved in communication. Note that these authors also implicated another X-chromosome neuroligin, *NLGN3,* in autism, but this is located at Xq13, where one copy would be inactivated, and there is no homologue on the Y-chromosome. Therefore, unlike *NLGN4*, *NLGN3* would not be over-expressed in those with an extra X or Y chromosome.

For the reasons described above, we may hypothesise that an extra copy of *NLGN4* could be implicated in neurodevelopmental problems. However, we also need to explain within-karyotype variation. Although there is a substantial increase in rates of speech, language and social communication problems in children with SCTs, the additional chromosome does not cause language impairment or ASD in a deterministic fashion. A minority of children have no evidence of developmental difficulties, a minority are severely affected with disabilities extending across many domains, and most have mild to moderate impairments ( Linden & Bender, 2002).

The wide variation in outcomes suggests that the extra gene dosage could act as a multiplier of other risk factors, which interact with the sex chromosome genes in a dosage-dependent manner and so only assume importance in the subset of individuals who have other genetic or environmental risk factors ( Bishop & Scerif, 2011). This explanation is consistent with rodent research comparing the effect of a *NLGN3* mutation between different strains of mouse, suggesting the impact is dependent on the genetic background ( Jaramillo *et al*., 2018). It also is compatible with evidence from studies of mutations in *NLGN4* in humans, which found that the same mutation may be associated with different phenotypes within one family ( Jamain *et al*., 2003; Laumonnier *et al*., 2004; Lawson-Yuen *et al*., 2008; Yan *et al*., 2005). As well as autism, *NLGN4* associations have been described with intellectual disability, language disorder and Tourette syndrome ( Lawson-Yuen *et al*., 2008; Yan *et al*., 2005).

## Hypothesis

Our pre-planned analysis was designed to test the ‘double hit’ hypothesis

### The ‘double hit’ hypothesis: Neuroligins act as a multiplier of effects of neurexins

**Figure 1.**
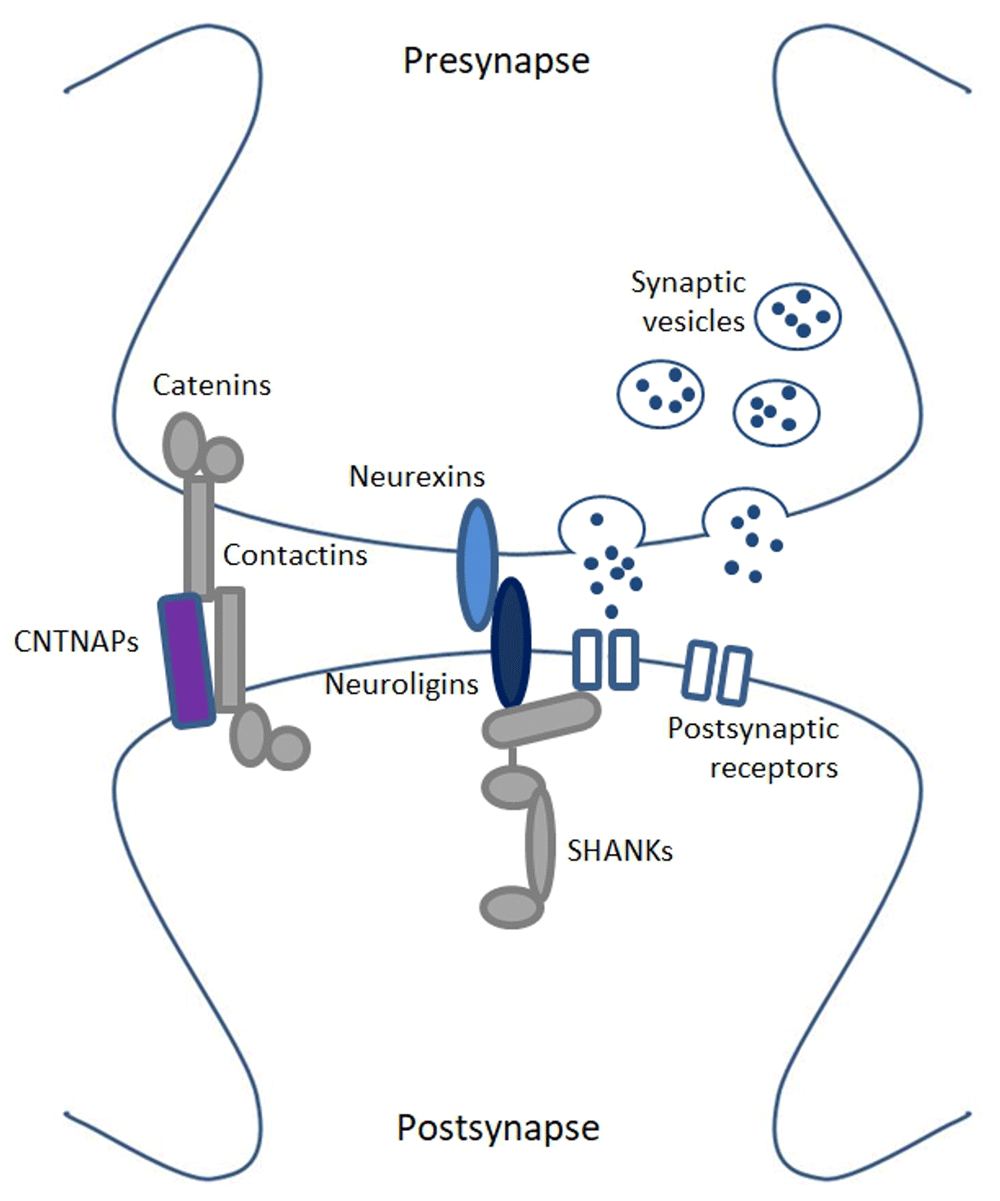
Neurexins (such as *NRXN1*), neuroligins (such as *NLGN4*) and contactin-associated proteins (such as *CNTNAP2*) all form part of the synaptic scaffolding system.

The notion of a ‘double hit’ aetiology has been proposed previously to account for cases where a microdeletion is inconsistently associated with neurodevelopmental disorder ( Girirajan *et al.*, 2010; Newbury *et al*., 2013): the idea is that a severe phenotype may be seen when there are two copy number variants or mutations, each of which may be relatively innocuous on its own. Here, we extend that idea to argue that the effect of altered *neuroligin* gene dosage may depend on the genetic background provided by autosomes ( Bishop & Scerif, 2011). In this regard, it is of particular interest to note that neuroligin proteins form part of the same functional network as a group of presynaptic transmembrane proteins, known as neurexins; their interactions play a key role in synaptogenesis ( Hussain & Sheng, 2005). *CNTNAP2* encodes a member of the neurexin superfamily whose polymorphisms have been associated with common forms of language impairment ( Graham & Fisher, 2015), though the effect size is relatively small ( Vernes *et al*., 2008). The role of the CNTNAP2 protein in developing brain is not fully understood, and it is likely to play multiple roles at different time-points. While early functional studies of the CNTNAP2 protein indicated that it localises to nodes of Ranvier in axonal membranes, it is now recognised to have key functions at the synapse ( Lu *et al*., 2016; Zweier *et al*., 2009). This raises the possibility that a *CNTNAP2* gene variant that has a modest effect in individuals of normal karyotype might have a much larger impact in the context of overexpression of a *neuroligin*. This hypothesis predicts that presence of an additional sex chromosome will amplify the impact of common genetic variants that have two characteristics: (a) they have been associated with DLD or ASD, and (b) they are in the same functional network as *neuroligins*. Figure 1 is a schematic showing two genes of interest to our current study, *CNTNAP*s and Neurexins, interacting with neuroligins in the synaptic cleft.

## Methods

We report how we determined our sample size, all data exclusions, all manipulations, and all measures in the study ( Simmons *et al*., 2012).

### Power analysis and impact of ascertainment bias

We aimed to recruit sufficient children with trisomies to detect an effect size of d = 0.5 for each copy of a given genetic variant on a phenotype, equivalent to a standardized regression slope of 0.25. The anticipated effect size is hard to judge, but the average impact of a sex chromosome trisomy on verbal IQ is more than one SD from the general population mean ( Leggett *et al*., 2010), suggesting that if the trisomy acts as a multiplier of effects of autosomal variants, this effect could be large. When testing variants with a prior association with disorder, we can make a directional prediction. We aimed to recruit 150 children with trisomies, which would have given 94% power to detect a slope of 0.25 on one-tailed test. However, we recruited only 140 children and had missing data on some variables, so numbers, and consequently power, are lower than this. In addition, we have to take into account that the sample is not representative of children with sex chromosome trisomies, because around 50% had the trisomy discovered in childhood when developmental difficulties were being investigated (see below). We devised a simulation to check the impact of these factors on power (see Appendix 1). This showed that a combination of N = 130 with 50% postnatally identified (and presumably biased) cases with mean phenotype score 0.9 SD below the group average (computed from a language factor score), reduced power to 87% on one-tailed test.

### Participants

**Figure 2.**
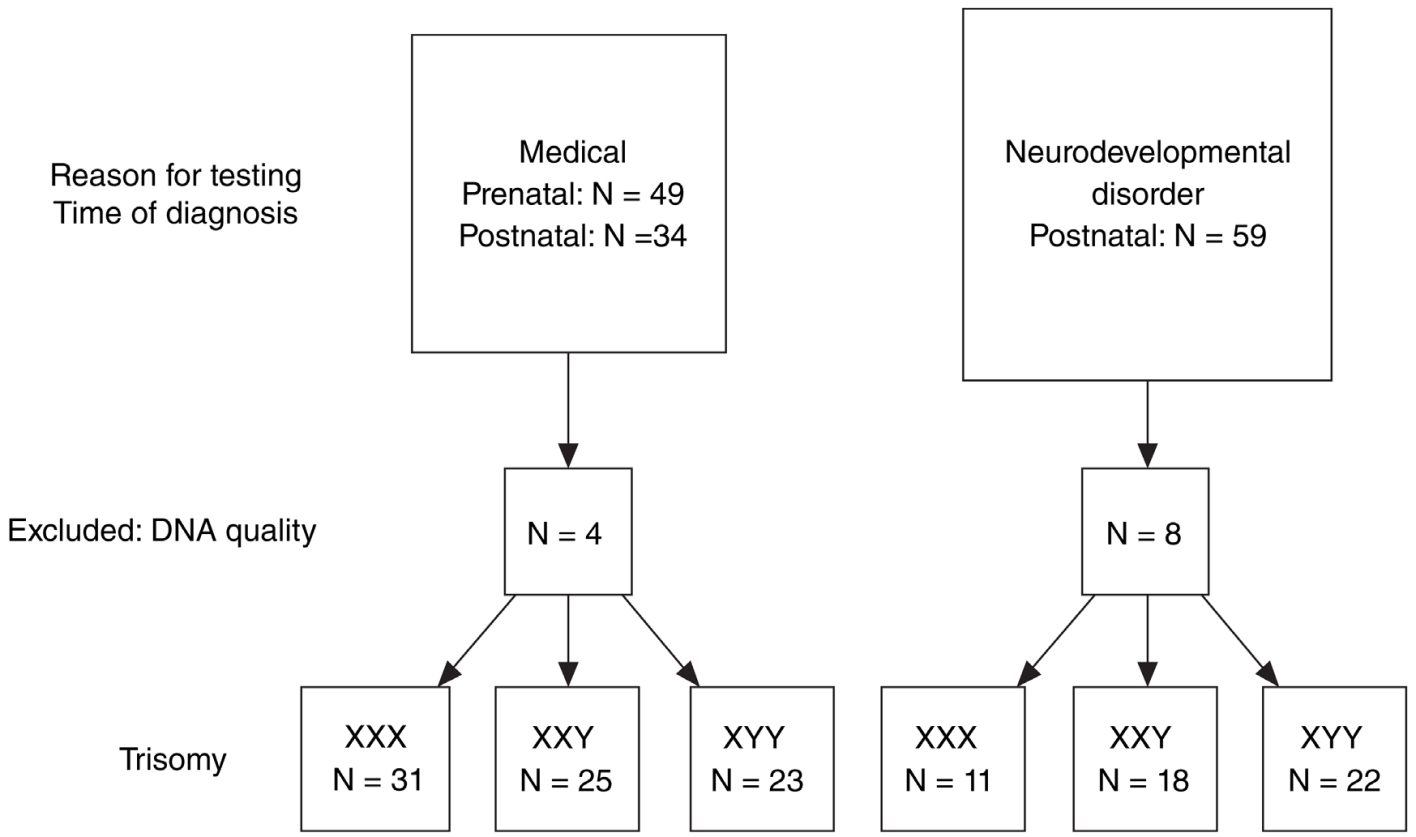
Flowchart showing characteristics of children recruited to sex chromosome trisomies (SCT) group.

Sex chromosome trisomies: After excluding children with missing or inadequate DNA, participants included 42 girls with trisomy X, 43 boys with Klinefelter syndrome, and 45 boys with XYY. These were combined in a single group of 130 children for analysis, but are shown broken down by trisomy and background in Figure 2. Cases were recruited from National Health Service Clinical Genetics Centres, from two support groups ( Unique: the Rare Chromosome Support Group, and the Klinefelter Syndrome Association), or from self-referral via advertisements on the OSCCI website and our Facebook page. A criterion for inclusion was that the child was aware of their trisomy status. In a previous study ( Bishop *et al*., 2011) we noted that levels of impairment tended to be lower in cases where the trisomy was discovered on prenatal screening than in those identified later in childhood. We therefore asked parents specifically about the reason for genetic testing; for 59 children aneuoploidy only came to light because of behavioural or developmental problems. Note that this means that data from this sample should not be used to estimate prevalence of neurodevelopmental disorders in sex chromosome trisomies.

**Figure 3.**
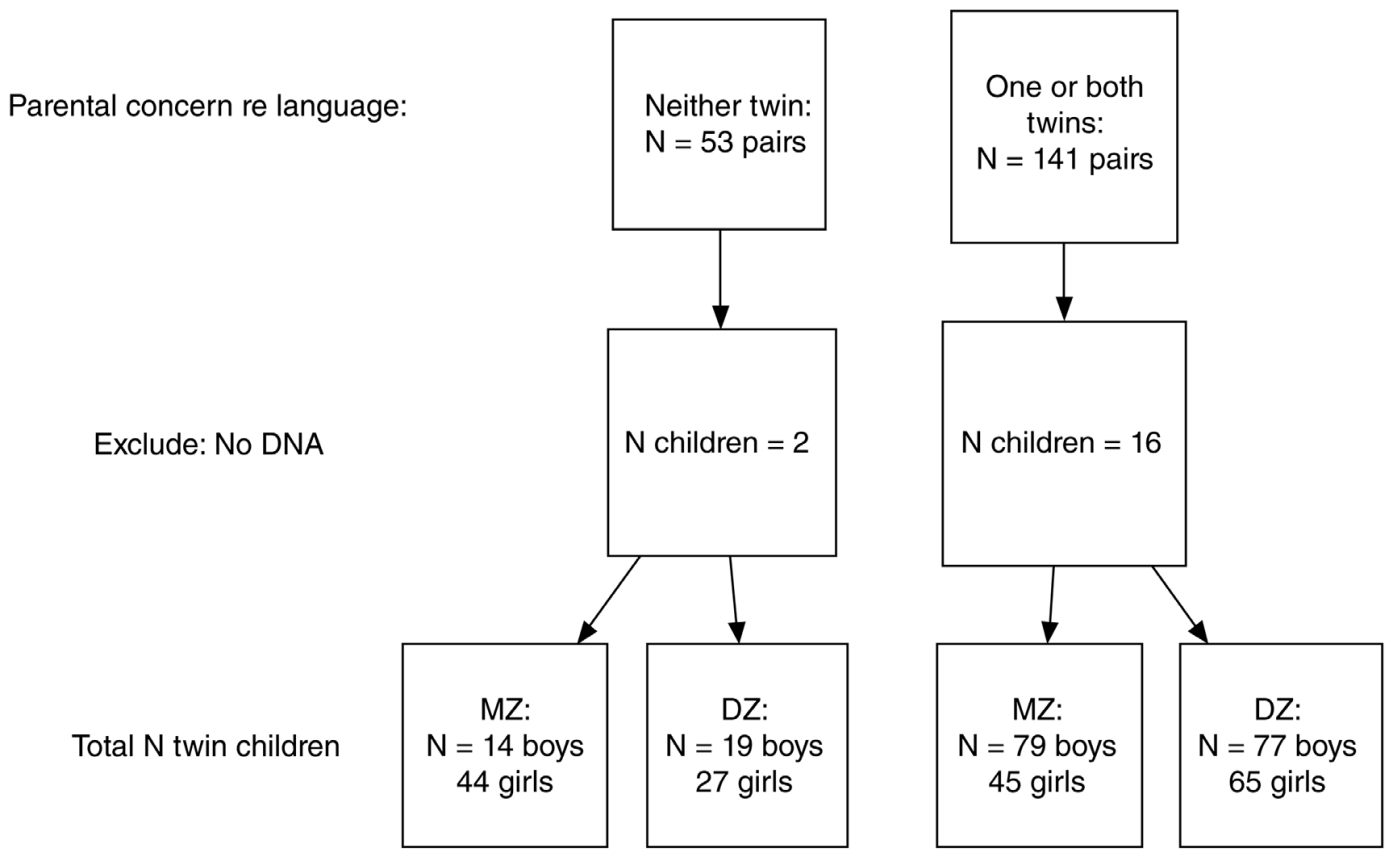
Flowchart showing characteristics of children recruited to comparison groups.

Comparison group: Comparison data came from a sample of children aged from 6 years 0 months to 11 years 11 months who had completed the same test battery, who were taking part in a twin study of language and laterality ( Wilson & Bishop, 2018a), and whose first language at home was English. Although twinning is a risk factor for early language delay, this effect appears to wash out with age, and by school age, genetic factors play a major role in the aetiology of language disorder ( Bishop, 2006; Rice *et al*., 2018). In this sample, we aimed for an over-representation of twin pairs in which one or both twins had language or literacy problems that might be indicative of DLD. This was coded on the basis of parental response on a telephone interview: any mention of language delay, history of speech and language therapy, current language problems or dyslexia was coded as ‘parental concern’. We aimed to recruit 180 pairs selected on the basis of having language or literacy problems (60 MZ, 60 DZ opposite sex and 60 DZ same sex), and 60 unselected pairs (20 of each type): we fell short of this goal as seen in Figure 3. For the current analysis, we grouped together all twins, regardless of zygosity and parental concern, and then divided them into two subsamples by selecting one twin from each pair at random, after excluding 18 cases with missing or insufficient DNA. This means we can replicate the analysis for twins with a diploid (typical) karyotype. Note that this replication sample is not independent, as the genotype for the MZ twins is the same in the two subsamples, and is related for DZ twins.

Information about zygosity, gender and parental concern is shown for information, but was not used in the analysis. Because twins are not independent, the final sample was divided into two subgroups of 184 and 186 children respectively, each containing one member from each pair, selected at random. (Ns not equal because some twins had missing DNA from just one member of the pair).

Some twin children had evidence of autism spectrum disorder (N = 15) or intellectual disability (N = 3), and twelve failed a hearing screen on the day of testing, although none of them had any known sensorineural hearing loss. For the current study, because we were interested in a broader phenotype than pure DLD, these cases were retained in the sample.

### Test battery

***Psychiatric evaluation.*** In an initial telephone interview, parents were asked about the child’s medical and educational history, including a question about whether anyone had diagnosed the child with a neurodevelopmental disorder such as ASD, developmental language disorder (DLD) or specific language impairment, dyslexia or dyspraxia. In addition, one or both parents were asked to complete the online Development and Wellbeing Assessment (DAWBA) ( Goodman *et al*., 2000) in their own time. 84 parents of SCT cases and 133 parents of twins complied with this request. The DAWBA gives information on likelihood of the child meeting criteria for a range of psychiatric diagnoses; a final diagnosis is made by a trained rater who assimilates all the information and evaluates it against DSM5 criteria ( American Psychiatric Association, 2013).

***Language, literacy and cognitive assessments.*** All children were seen at home or in a quiet space in their school for a neurocognitive assessment, using the battery of language and nonverbal ability tests shown in Table 1. Hearing was screened in left and right ears using a DSP Pure Tone Audiometer (Micro Audiometric Corporation). The child was familiarised with the task of raising their hand on hearing a tone using 40 dB (HL) tones. They were then tested with 25 dB pure tones at frequencies of 500, 1000, 2000 and 4000 Hz. Louder tones were presented in 5 dB steps to establish a threshold at any frequency where a 25 dB tone was not detected. Children with an average threshold greater than 30 dB in the better ear were categorized as failing the screen. The battery also included tests of literacy: the Picture and Digit naming tests from the Phonological Assessment Battery ( Frederickson *et al*., 1997), the Test of Word Reading Efficiency ( Torgesen *et al*., 1999) and the Neale Analysis of Reading Ability -2 ( Neale, 1999), but these are not included in the current analysis as there was much missing data from the youngest children. In addition, handedness and language laterality were assessed. Results from laterality assessments were unremarkable and are not considered further here ( Wilson & Bishop, 2018a; Wilson & Bishop, 2018b).

**Table 1.** Assessment battery.

Instrument	Measure
Woodcock Johnson III Tests of Cognitive Abilities ( Woodcock *et al*., 2007)	Verbal Comprehension
NEPSY: A Developmental Neuropsychological Assessment ( Korkman *et al*., 1998)	Repetition of Nonsense Words
	Oromotor Sequences
	Sentence Repetition
Wechsler Abbreviated Scale of Intelligence (WASI) ( Wechsler, 1999)	Vocabulary
	Block Design
	Matrices
**Parental questionnaires**	
The Children’s Communication Checklist-2 (CCC-2) ( Bishop, 2003)	
The Social Responsiveness Scale (SRS) ( Constantino, 2005)	

For the NEPSY tests, norms extend only to age 12 yr 11 months, and so we used extrapolated scores, as documented in Appendix 2.

### Phenotypes

We considered three quantitative phenotypes ranging from a specific measure of a heritable language skill, through a more general language measure, to a measure that potentially indexes a wide range of neurodevelopmental problems:

A) Nonword repetition, which is regarded as a measure of phonological short-term memory. This was singled out as an individual measure because it has previously been identified in twin studies as a good marker of heritable language problems ( Bishop *et al*., 1996) and has also been associated with genetic variants linked to language/literacy in the *CNTNAP2*, *CMIP*, *ATP2C2*, *KIAA0319*, and *DCDC2* genes ( Carrion-Castillo *et al*., 2017; Marino *et al*., 2012; Newbury *et al*., 2009; Newbury *et al*., 2011; Scerri *et al*., 2011; Vernes *et al*., 2008). In the current study, we used scaled scores from Repetition of Nonsense Words from the NEPSY ( Korkman *et al*., 1998).
B) A general language factor derived from the four other language tests (Verbal Comprehension, Oromotor Sequences, Sentence Repetition and Vocabulary). As documented in Appendix 3, the decision to combine these measures into a single language factor was made after exploring the factor structure of the available phenotypic measures, with the goal of obtaining a reliable indicator of overall language function.
C) A global measure of burden of neurodevelopmental problems extending beyond language, including autistic features. This was developed on an *ad hoc* basis, using all available information from parental report (see Appendix 4).

### DNA collection and analysis

Oragene kits (OG-500, DNA Genotek Inc, Ontario, Canada) were used to collect saliva for DNA analysis from children with SCTs and their parents and available twin pairs. DNA extraction was performed using an ethanol precipitation protocol as detailed in the standard protocol (DNA genotek). All extracted DNA was genotyped on the Infinium ‘ Global Screening Array-24 (v1)’, which includes 692,824 SNPs including rare and common variations. Data were processed in the Illumina BeadStudio/GenomeStudio software (v. 2.03) and all SNPs with a GenTrain (quality) score of < 0.5 were excluded at this stage. All genotypes were further filtered using PLINK software v1.07 ( Purcell *et al*., 2007); as recommended by Anderson *et al*. (2010), samples with a genotype success rate below 95% or a heterozygosity rate ±2 SD from the mean were removed, as were SNPs with a Hardy-Weinberg equilibrium P < 0.000001 or a minor allele frequency of less than 1%. Identity data within families and twin-pairs were used to exclude samples with unexpected gender or relationships. SNPs that showed an inheritance error rate > 1% or skewed missing rates between genotype plates were also excluded. Control data (CEU, YRI, CHB, JPT, Hapmap release #3) were employed through a principal component analysis within Eigenstrat ( Price *et al*., 2006) to identify individuals with divergent ancestry. Sixteen individuals (6 twin pairs and 4 SCT cases) were identified as having African ancestry and 21 individuals (6 twin pairs and nine SCT family members) were identified as having Asian ancestry. Any SNPs that showed a significant association with non-European ancestry (P < 0.0001) were excluded. The final genome-wide dataset consisted of 500 individuals (370 twins, divided into two subgroups, and 130 independent SCT cases) and 451,093 autosomal SNPs with a genotyping rate of 99.78%.

### Procedure

Ethical approval was obtained for the study in 2011 from the Berkshire NHS Research Ethics Committee (reference 11/SC/0096), and data collection started in August of that year, finishing in October 2016. Information sheets, consent forms and ethics approval documents are available on Open Science Framework. Families who had expressed interest in the study were interviewed by telephone to assess whether the child met inclusion criteria, and if so, an appointment was made to see the child at home or at school, depending on parental preference. Families were widely dispersed around the UK, including Northern Ireland, Scotland, Wales and England. During the course of recruitment a total of eight research assistants as well as the senior author were involved in assessing children. The assessment was conducted in a single session lasting between 2–3 hours per child, with breaks where needed.

## Analysis plan

Study data were analysed using R software ( R Core Team, 2016), with the main database managed using REDCap electronic data capture tools hosted at the University of Oxford ( Harris *et al*., 2009).

Potentially, there is a very large number of genotypes and phenotypes that could be analysed to test our hypothesis, as well as different ways of creating subgroups. This consideration, coupled with the small sample size, makes it important to control adequately for multiple testing to guard against type I error ( Grabitz *et al*., 2018). For this reason, we stored phenotype and genotype data separately and specified an analysis plan in detail, as reported in our stage 1 registered report ( Newbury *et al.,* 2018). The analysis of genotype-phenotype associations was conducted after this plan had been registered and peer-reviewed.

### Subgroups

In our main pre-specified analysis we treated all three trisomies together. This is because the double hit hypothesis postulates a common mechanism that would apply regardless of karyotype. We specified that if we found an association between genotype and phenotype, we would carry out exploratory analyses to consider whether this is moderated by karyotype. This would allow us indirectly to test a prediction by Skuse (2018) that there is more variable expressivity of *NLGN4X* than *NLGN4Y*. If so, one might expect a more severe impact of a double hit in the XYY group, which would be reflected in the epistatic interactions with NLGN. and should lead to lower phenotypic variability in XYY compared to the other karyotypes. Note, however, that the ascertainment bias in the sample is problematic for making cross-karyotype comparisons, and the focus would have to be just on those who were not diagnosed because of neurodevelopmental problems (see Figure 2). This is a small sample and so there would be a high risk of missing a true effect (type II error).

### Prioritising genotypes for analysis

We conducted a series of literature searches to prioritise autosomal genes for analysis, focusing on genes that had an association with childhood speech and language disorders and that were relevant for synaptic function (see Appendix 5). This led us to select two candidates; *CNTNAP2* and *NRXN1*. Both of these genes are large (>1 MB) and included over 100 SNPs from the genotyping array. In order to avoid false positives with our small sample size, we chose to focus our analysis on regions that have previously been associated with neurodevelopmental disorder, analysing all genotyped SNPs (after quality control steps described above (see “DNA collection and analysis”) within these selected regions.

In *CNTNAP2* ( NM_014141), we focused on a region spanning exons 13–14 (chr7:147,514,390-147,612,852 (hg19)). This region includes a cluster of 9 SNPs previously associated with language disorder ( Vernes *et al.*, 2008; Whitehouse *et al*., 2011). We had direct genotype data for 22 SNPs across this region. In addition, we used imputation to obtain genotypes for SNPs rs2710102 and rs7794745. These were the first SNPs reported to be associated with ASD, and represent the two main SNPs used in the majority of association studies in neurodevelopmental disorders ( Alarcón *et al*., 2008; Arking *et al*., 2008). These two SNPs were not directly genotyped on the Illumina arrays and were therefore imputed for all individuals. Imputation was performed on the Michigan Imputation Server, an online server which generates phased and imputed genotypes using high-density reference panels. Variant Call Files (VCF) were uploaded for 15,936 SNPs genotyped on chromosome 7. Genotypes were phased within Eagle and imputed by Minimac against the Human Reference Panel hrc.r1.1.2016, which includes 64,940 haplotypes of predominantly European ancestry. In total, genotypes were generated for 2,289,829 SNPs across chromosome 7, 513,970 of which had quality scores 0.9. The two SNPs of interest, rs2710102 and rs7794745 had quality scores of 0.9938 and 0.94127 respectively.

The second candidate is *NRXN1* ( NM_004801). Although this gene met our criteria of being relevant for both synaptic function and neurodevelopmental phenotypes, the studies showing this link involved deletions rather than common variants ( Ching *et al*., 2010). A recent analysis of clinical microarray data showed that deletions in exons near the 5′ end of *NRXN1* were specifically implicated in neurodevelopmental disorders ( Lowther *et al*., 2017). Accordingly, we focused on 23 SNPs in this region of the gene chr2:51,141,501-51,280,121 (hg19). These SNPs covered exons 1–4 plus 20 Kb upstream (5’) of the gene as this region includes important regulatory sequences. Details of the SNPs included in the analysis are shown in Appendices 6 and 7.

The SNPs within the chosen regions were filtered for minor allele frequency and Hardy Weinberg Equilibrium (as outlined in DNA collection and analysis) but were not pruned for linkage disequilibrium. Previous simulations indicate that the Generalized Structured Component Analysis (GSCA) method is not greatly affected by linkage disequilibrium (see Romdhani *et al.*, 2014). Across the *CNTNAP2* region, six pairwise combinations of SNPs had R2 > 0.8. Across the *NRXN1* region, 8 pairwise combinations of SNPs had R2 > 0.8. Details of the SNPs included in the analysis and a table of correlations between SNPs are shown in Appendix 8.

### Statistical methods

**Figure 4.**
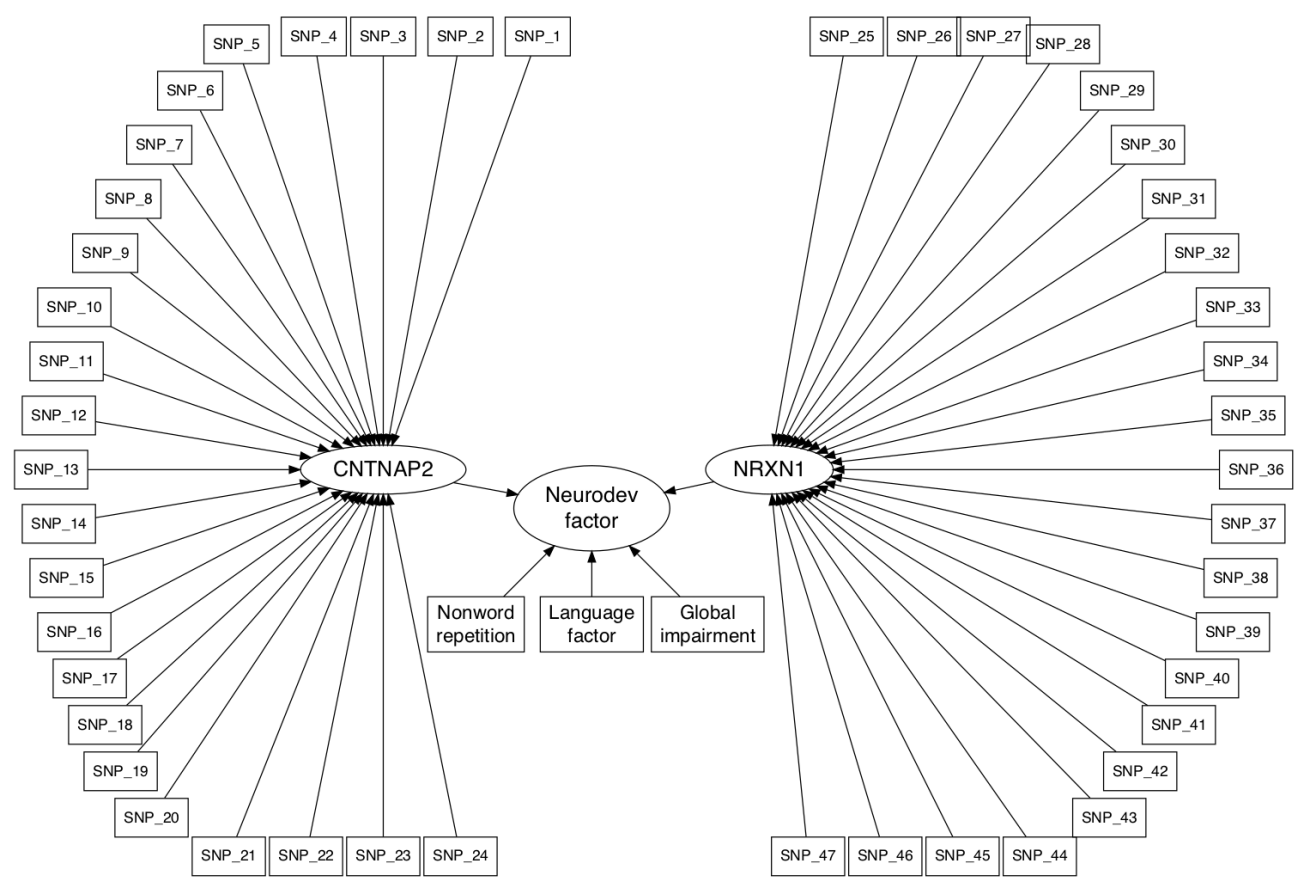
Structural equation diagram for analysis.

*CNTNAP2* and *NRXN1* genes from the trisomy sample were analysed for association with a latent variable based on the three phenotypes using a structural equation modelling (SEM) approach adapted for genetic analysis ( Romdhani *et al*., 2015). The model specification for our analysis is shown in Figure 4.

The GSCA analysis estimates the path from each gene to the Neurodev factor, with significance calculated by permutation analysis.

Romdhani *et al*. (2015) used the GSCA developed by Hwang & Takane (2004). This method uses component-based path modelling rather than traditional covariance-based SEM, allowing adequate model fit to be achieved when using smaller samples ( Chin & Newstead, 1999; Tenenhaus *et al*., 2005). The measurement models in the SEM framework are not typical regression format using latent factors; instead they are fitted using alternating least squares to estimate the weights and parameters, which is similar to principal components analysis. The advantage of this approach is that it does not attempt to fit the whole covariance matrix for observed and latent variables, but rather fits a separate measurement model for the contribution of observed variables to each latent factor, as well as a covariance model for the latent factors. Hence, we do not estimate the contribution of individual SNPs in each gene to the phenotype; rather, their influence is represented via the weighted sum. Similarly, the latent phenotypic factor (termed Neurodev factor in Figure 4) is a weighted sum of the three measures of the phenotype. We estimated the significance of one direct pathway from the *CNTNAP2* gene to the latent phenotype, and one from *NRXN1* to the latent phenotype. This method thus gives a single estimate of the overall impact of SNPs in a region of the phenotype.

We conducted simulations that indicated that this method is feasible with the number of SNPs and phenotypes in our sample (see Appendix 1): the permutation method, used by this approach to effectively quantify the test statistic distribution, generates p-values independently for each path, and a correction is required to take this into account. Because the evidence of association of common variants was stronger for *CNTNAP2* than for *NRXN1*, we used a sequential approach to setting a significance level (alpha), using a critical p-value of .05 to test the pathway from *CNTNAP2* to the Neuro factor, and .025 for the pathway from *NRXN1* to the Neuro factor.

In addition, we conducted the same analyses with children from the two comparison samples.

We predicted that one or both paths from *CNTNAP2* and *NRXN1* to the Neurodev factor would indicate significant association in the sex chromosome trisomy sample. We further predicted that any associations in the comparison samples will be similar in direction, but smaller in size and may not reach statistical significance.

### Further exploratory analyses (update added to version 3 of paper)

***Gene-based analyses*.** Following the publication of the GSCA analyses described above, we performed gene-based analyses of the 22 candidate genes described in our analysis plan ( Appendix 5, Figure S1). These methods simultaneously consider genetic variation across a gene unit in a single individual, incorporating both risk and protective effects. These effects are considered in a cumulative model in which a higher number of risk variants is associated with poorer outcome at the behavioural level. Such analyses can handle large numbers of variants and allow us to include variants that are individually rare. Note, however that gene-based approaches are still limited to consideration of single genes and often involve many parameters, increasing the potential for false positives. This approach is an exploratory analysis that may lead to the identification of a functional targeted subset of variants for further investigation. If rare variants were found to have an effect, then these could be followed up using GSCA analysis ( Thompson *et al.*, 2020) as performed for *CNTNAP2* and *NRXN1* here.

Quantitative association analysis was performed for common and rare variants within the 18 autosomal genes and four X chromosome genes identified in a literature search as having an association with childhood speech and language disorders and relevant synaptic function (Figure S1). Through an imputation step, these analyses included all known variants across the 22 candidate genes. We tested for association with each of the three outcome measures of interest; nonword repetition, language factor and neurodevelopmental factor.

Genotypes were imputed per chromosome using the Michigan Imputation server ( Das *et al.*, 2016) in the sex chromosome trisomy (SCT) samples and Twin sample sets. Genotypes from the Infinium ‘Global Screening Array-24 (v1)’ were extracted for the chromosomes containing the candidate genes and prepared for imputation in the following way. The Non-SNPs, (indels and multiallelic variants), A/T and G/C allele SNPs were excluded. Inconsistent reference alleles were also identified. These were then ‘forced’ so that the reference allele changed to the other allele or ‘flipped’ to the other strand, in PLINK v1.9. The vcf was then uploaded to the Michigan Imputation server using the following options; Human Reference panel - HRCr1.1 2016, Phasing - Eagle v2.3, Population - EUR, Mode - Quality Control and Imputation. For chromosome X all the options were the same apart from the Phasing - ShapeIT v2.r790 (unphased) as recommended by the Michigan Imputation Server. This reference panel consists of 64 976 haplotypes of predominantly European ancestry for 32 488 individuals across over 39 million variants genomewide. 

For each candidate gene, as annotated in hg19, the imputed SNPs were extracted for that region +/- 10kb either side. SNPs that had an R2 (quality score) <0.7 in the Michigan Imputation server ‘.info’ file were excluded, as were SNPs that appeared twice in the output (multi-allelic variants). The allele frequencies were checked and monomorphic SNPs were excluded. Hardy Weinberg Equilibrium was checked in vcftools and any SNPs with p<10 ^-6^ were excluded. Plink v1.9 was used to identify Mendelian errors in the SCT sample set, for whom parental genotypes were available. For samples with Mendelian errors individual genotypes were removed. Genotype rates were 100% per SNP following imputation. Appendix 10 lists the number of SNPs analysed per gene for each sample set and test.

Tests of association with the three quantitative phenotypes: nonword repetition, language factor and global neurodevelopment index, were performed using Rvtests ( Zhan *et al.*, 2016) in the SCT and Twin sample sets. For rare variants we used the Zeggini test ( Morris & Zeggini, 2010) which is an aggregation test suitable for variants with MAF < .01. It computes a cumulative score of rare variant burden across the gene of interest which is then tested for association to a phenotype. The four X chromosome genes were excluded from the analysis for the SCT sample as genotypes did not follow conventional identification.

In a further analysis we used the SKAT test, which handles both common and rare variants within a single test (MAF upper limit 0.5). Both the Zeggini and the SKAT tests group the variants into a unit, in this case a gene.

## Results

**Figure 5.**
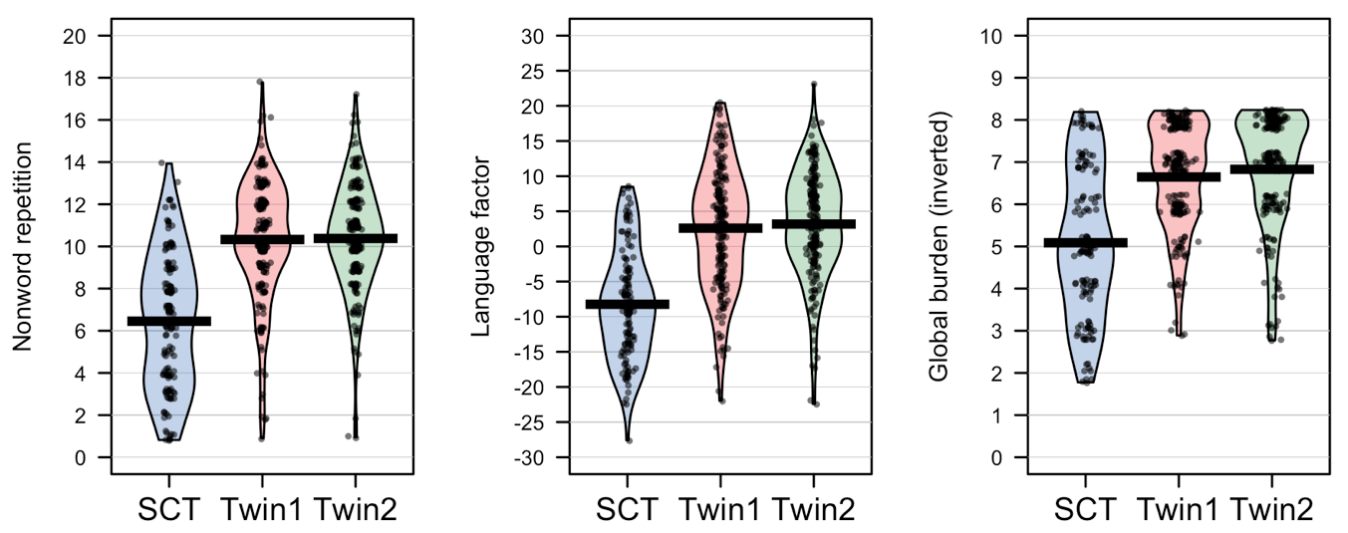
Pirate plots ( Phillips, 2017) for sex chromosome trisomies (SCT) and two twin groups, showing individual cases as points, with bold line depicting median, for Nonword repetition (scaled score), Language factor and Global neurodevelopmental impairment (inverted so low score reflects impairment).

Figure 5 shows the distributions of scores on the three phenotypes for children with sex chromosome trisomies and the two comparison groups. The scores for Global burden are inverted so a low score corresponds to impairment, to be consistent with the other two measures, and scores on Global burden and Nonword repetition are jittered vertically as well as horizontally for clarity. Phenotypic characteristics of children with SCTs will be the focus of a separate publication, but we may note that, as anticipated, the group with SCTs show evidence of impairment on all three phenotype measures, but with a wide range of scores. For the combined sample with all cases (N = 500), nonword repetition correlated 0.76 with the language factor, and 0.60 with the global impairment rating. The language factor and global impairment rating correlated 0.69.

### GSCA path-fitting analysis

Data from the three phenotypes were fitted using the model in Figure 4, first for the SCT group, and then separately for the two comparison groups. Table 2 shows the p-values based on 5000 permutations. The association with the ‘Neurodev’ factor did not meet our criterion for significance for either *NRXN1* nor *CNTNAP2* (see Table 2).

**Table 2.** P-values from 5000 permutations for association with neurodevelopmental factor for *NRXN1* and *CNTNAP2*: results from Generalized Structured Component Analysis (GSCA).

Group	*NRXN1*	*CNTNAP2*
SCT	0.703	0.268
Twin1	0.080	0.323
Twin2	0.162	0.524

### Exploratory analyses

Exploratory analyses were conducted to rule out the possibility that our analytic approach may have missed significant associations with specific SNPs. Appendix 9 shows associations computed for all the individual SNPs entered into the analysis, as well as the regression slopes relating number of minor alleles to phenotype for the two SNPs in *CNTNAP2* that have been a particular focus of research attention. The distribution of p-values seen for the whole collection of SNPs did not differ from that expected by chance.

In addition, we considered the suggestion by Skuse (2018) that results may vary by karyotype, because the expression of the X-linked homologue ( *NLGN4X)* is variable, whereas in males with XYY, *NLGN4Y* is fully expressed. Although we did not assess *NLGN4X* and *NLGN4Y* directly, one might expect a more severe impact of a double hit in the XYY group, which would be reflected in the epistatic interactions with *NLGN*. To test this, we reran the GSCA separately for XXX, XXY and XYY subsamples, restricting consideration to those who were diagnosed prenatally, as specified in our protocol. As shown in Table 3, there was no hint of any association between genotype and phenotype in these subgroups. Note, however, as we originally stated, this analysis has very low power to detect true effects.

**Table 3.** P-values based on 5000 permutations for association with neurodevelopmental factor for *NRXN1* and *CNTNAP2* for sex chromosome trisomies (SCT) cases subdivided by karyotype, excluding those identified because of neurodevelopmental problems: results from Generalized Structured Component Analysis (GSCA).

Group	*NRXN1*	*CNTNAP2*
XXX, N = 31	0.311	0.256
XXY, N = 25	0.341	0.076
XYY, N = 23	0.787	0.447

### Further exploratory analyses added in version 3

**Gene-based analyses.** After Bonferroni correction for multiple testing none of the p-values were significant across the 22 candidate genes tested ( Appendix 10) ( Bishop *et al*., 2019).

## Discussion and conclusions

We assessed a “double-hit” model of differential susceptibility in children with sex-chromosome trisomies (SCTs), focusing on the hypothesis that the effects of common variations in neuroligin/neurexin genes upon language development are amplified in children with a sex chromosome trisomy ( Bishop *et al*., 2011). This hypothesis was evaluated through a targeted analysis of two genes in the neuroligin/neurexin pathway, *CNTNAP2* and *NRXN1*, both of which have previously been associated with language development. A set of 47 SNPs were divided into corresponding candidate genes forming latent factors (weighted sums, in this case). These were analysed in relation to neurodevelopmental outcomes in a cohort of children with sex chromosome trisomies and two comparison twin groups, within a generalised structured component model ( Hwang & Takane, 2004). Outcomes were represented by three related latent phenotype factors; non-word repetition, a measure of phonological short-term memory that has previously been associated with genetic variants associated with language and/or literacy, a general language factor score, and a measure of global neurodevelopmental impairment.

Children with SCTs showed evidence of impairment across all three of these phenotypes, indicating that they represent sensitive markers of the difficulties encountered by children with SCTs, and there was a wide range of severity of impairment. However, a factor derived from the three phenotypes was not associated with either candidate gene within the SCT sample or the two comparison datasets. Permutation testing confirmed that no effects reached our specified level of significance.

To address a concern that our method may mask associations with specific SNPs, we conducted an exploratory investigation of the individual variants within *NRXN1* and *CNTNAP2* (see Figure 4) using a more conventional regression approach testing for association with each of the three outcome factors ( Appendix 9). The distribution of p-values across SNPs within each of the groups, aligned with that expected by chance, taking into account the correlations within the SNP set and the correlations between phenotypes.

**Figure 6.**
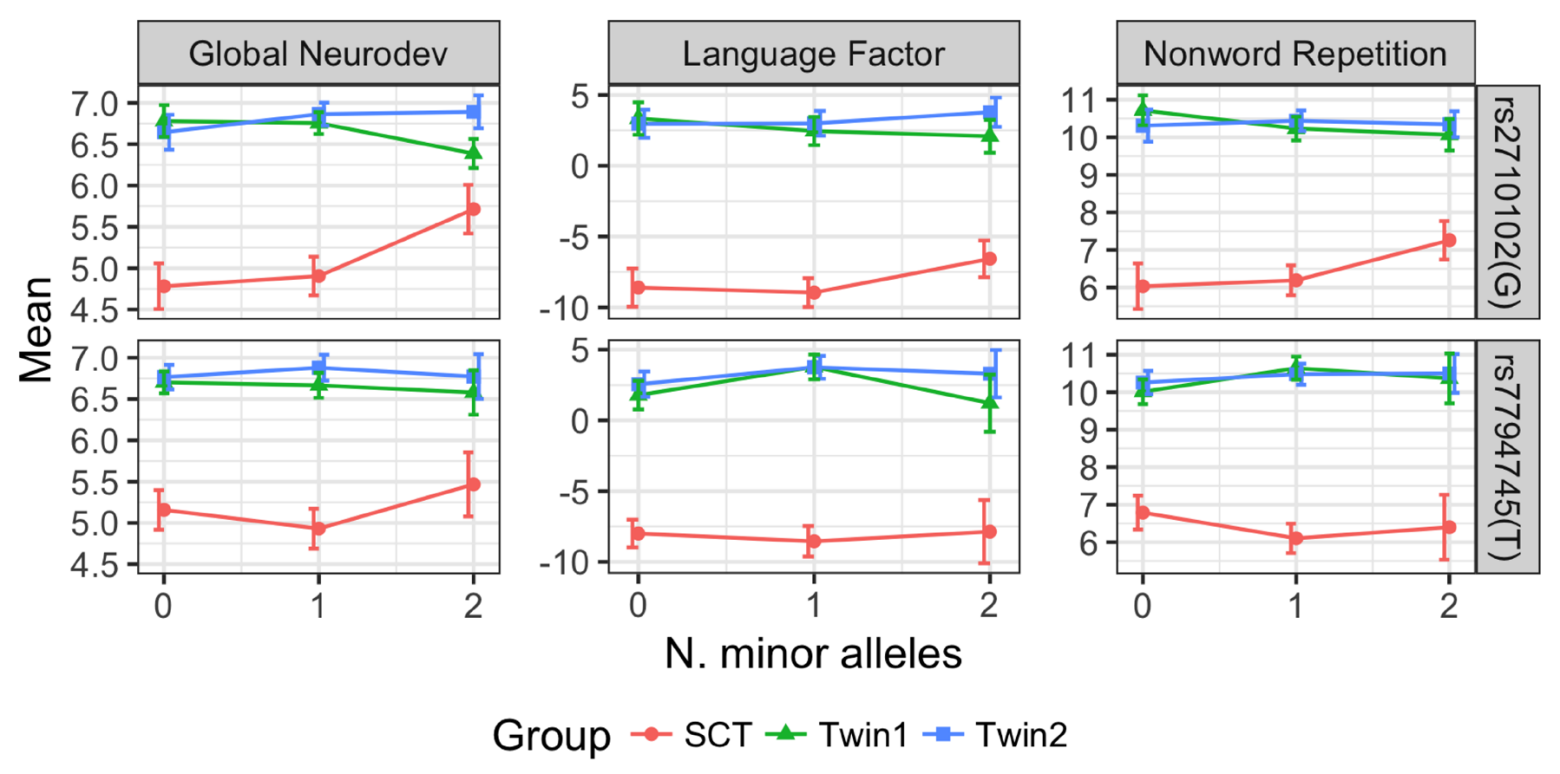
Mean scores for three phenotypes for SCT and both twin groups, in relation to number of minor alleles for rs2710102(G) and rs7794745(T).

Figure 6 shows results for two variants in *CNTNAP2* that had previously been robustly associated with language (rs779475 and rs207102). Previous studies indicate that for both of these SNPs the minor allele (T allele for rs779475 and G allele for rs207102) was associated with poorer neurodevelopmental outcomes ( Alarcón *et al*., 2008; Vernes *et al.*, 2008; Whitehouse *et al*., 2011). However, in the current study, the direction of effects fluctuated between sample groups and outcome measures for both SNPs (see also Appendix 9) and did not approach statistical significance.

### Lack of association

Considering first the failure to replicate associations between specific SNPs and phenotypes in the twin comparison samples, we should not be surprised by this result. Non-replication is common in genetic association studies and primarily reflects the complexity of the underlying genetic effects ( Hirschhorn *et al.*, 2002; Ott, 2004). The majority of genetic contributions to language development are reported to have small effect sizes and are characteristically heterogeneous ( Newbury *et al.*, 2014). Our sample size gave limited power to detect the kinds of effect size obtained in previous studies.

For the SCT sample, we argued that effect sizes would be larger if the double hit hypothesis was correct. However, it was still the case that unless we took steps to maximise the chances of association and to guard against type I errors we were at risk of obtaining spurious results ( Grabitz *et al.*, 2018). This meant that we had to decide in advance which genes to focus on: it is entirely possible that a different selection of SNPs might have provided evidence for the double hit hypothesis: the challenge for this study was how to determine the optimal strategy for analysis. We used a structured literature review to identify genes implicated in developmental language disorder, generating a list of 41 candidate genes. Publication data for these genes were further scrutinised to narrow our search to a small number of genes with consistent and robust evidence for association. In this stage, we focused upon functional and behavioural dimensions of synaptic function and speech, language and communication respectively. Two genes scored highly on both of these dimensions and were selected for further literature-based review. One hundred and seven papers referring to *CNTNAP2* were examined. Of these, 32 included an analysis of common genetic variants and 19 examined rare variants of putative function. These papers tended to focus upon autism spectrum disorders but also considered speech and language and a diverse range of neurodevelopmental outcomes including intellectual disability and schizophrenia. Of the 32 common variant papers, 27 (84%) reported positive association, primarily with SNPs rs2710102 and/or rs7794745. The *CNTNAP2* gene has been implicated by both genome-wide screening approaches and targeted replication investigations and is reported to have a moderate effect size upon neurodevelopmental outcomes ( Vernes *et al.*, 2008). One hundred and thirty one *NRXN1* papers were examined, including 45 reporting rare variants and 20 investigating common variants. For the *NRXN1* gene, there was a clear focus upon the relevance of copy number variants (deletions and duplications) in autistic disorder, epilepsy and schizophrenia in the rare variant studies while investigations of common variants focused upon nicotine dependency and working memory. Interestingly, a recent analysis of clinical microarray data showed that deletions in exons near the 5’ end of *NRXN1* were specifically implicated in neurodevelopmental disorders ( Lowther *et al.*, 2017). Given the large size of these two genes, we chose to focus our analysis on regions that have previously been associated with neurodevelopmental disorder, namely exons 13-14 in *CNTNAP2* and the 5’ region of *NRXN1*. This evidence-based strategy reduced the possibility of false positives by providing a clear targeted rationale. However, it meant that our analyses were restricted to a small number of genetic variants within only two candidate genes. Within a complex genetic model, it is unlikely that one or two genes will explain a large proportion of the variance observed in language and communication, even under a hypothesis of increased susceptibility. Furthermore, our study design means that earlier identified candidates received more focus as they had a greater volume of supporting publications. We acknowledge that it is possible that we would have found association to alternative genes and/or SNPs if our search space had been extended but the chances of a type I error would also have increased ( Grabitz *et al.*, 2018). Further exploratory gene-based analyses of 22 genes, as presented in the updated sections, indicate that this search-space would have to be considerably increased to yield positive associations. As such, the publication of a pre-registered strategy helped us to consider effect sizes and analysis strategies upfront, balancing out these considerations before the analysis stages.

### Analysis methods

Genetic association studies typically consider each SNP as an independent variable giving rise to a particularly high number of tests. The typical significance threshold for a genome wide association study (GWAs) is 5×10 ^-8^ and a Bonferroni correction for the 47 SNPs and three factors tested in the current study stands at 0.00035. Such adjustments are largely considered to be overly conservative as they do not account for the relationships between variants or the increased density of available genetic information ( Fadista *et al.*, 2016). Recent developments have therefore been driven by gene-based analyses in which all variants within a gene are collapsed into a single factor for analysis. Gene-based methods reduce the number of tests required and can be applied to common and rare variants and allow for heterogeneous effects across a given gene ( Lee *et al.*, 2014). Many gene-based methods exist, but most make quite specific assumptions and different tests can provide optimal power depending on study design ( Lee *et al.*, 2014).

In this paper, we employed a gene-based analysis method which adapts a generalized structured component-based path model (GSCA) ( Hwang & Takane, 2004) for genetic analysis. Previous studies indicate that this method is sensitive within relatively small sample sizes and enables the consideration of multiple related variables within a small number of latent factors ( Romdhani *et al*., 2015). Our own simulations ( Appendix 1) indicated that this method was appropriate for a relatively small number of SNPs (<50) and traits, making it an attractive approach for a targeted hypothesis-led approach. Nonetheless, GSCA is a relatively novel approach to genetic analyses and the allowable number of variants remains small in genetic terms. More than 100 SNPs would be required to capture full information regarding common genetic variation across *CNTNAP2* and/or *NRXN1* and this dataset would be too large to give reliable results with our sample size.

Other gene-based tests based upon alternative statistical methods (e.g. burden tests, variance components and weighted analyses) are available and were applied to a limited number of candidate genes in our updated analyses. Even though these further exploratory investigations allowed a more comprehensive screening of genetic variation than the original analyses, which considered the effects of single SNPs, no significant associations were observed. These findings indicate that a much larger number of genes would need to be screened, which is not possible within the current dataset. As a general rule, the number of participants should be significantly greater than the number of parameters measured. Gene-based methods collapse effects across SNPs reducing the number of parameters to be estimated; however, adequate sample size should be ensured and not based on reducing dimensionality of data to achieve satisfactory power.

### Alternative models of effects

Our strategy focused upon common genetic variants within a small number of well-supported candidate genes but it is clear that language and communication are complex traits which involve many interacting loci with different functional effects. Many candidate genes have been identified and characterised in the context of severe neurodevelopmental disorders and large hit events (rare variants and/or copy number changes) and it is possible that the effects of a given risk variant may be affected by genetic background, the mechanism of mutation and environmental factors ( Weiner *et al.*, 2017). There are many possible mechanisms of functional interaction that would not be detected by the approach taken in this study. For example, variants which affect gene expression (eQTLs) typically occur outside of coding regions and can have long-range effects ( GTEx Consortium *et al.*, 2017). Since this study targeted restricted gene regions, we would not have detected long-range or trans- effects upon gene expression. Indeed, the control of gene expression is a complex process which involves many factors and this is complicated in this case by the involvement of X-inactivation and aneuploidy. Thus it would be of interest to directly measure the expression of the *NLGN4* genes and relate these to neurodevelopmental outcomes. Although this approach would only offer a snapshot of gene expression in time and space, it would be one step closer to contributory mechanisms and would provide a picture of the effects of aneuploidy upon gene expression and vice versa. Similarly, while a SNP-based analysis may tag structural rearrangements (deletions and duplications), an alternative approach would be required to fully assess the role of copy number variants. Copy number variants have been reported to play a role in neurodevelopmental disorder ( Girirajan *et al.*, 2011) and developmental language disorders ( Simpson *et al.*, 2015).

In summary, we did not observe association to either the *CNTNAP2* or *NRXN1* genes in our SCT cases or comparison controls, nor to a further 22 candidate genes in an extended analysis (version 3). We cannot reject the double-hit hypothesis on the basis of these analyses alone, but we can conclude that variants in the specific gene regions that we focused on do not appear to explain the phenotypic variation in neurodevelopment that is seen in children with an additional sex chromosome. In order to further the double hit hypothesis, additional analyses that consider many more candidate genes and/or different functional mechanisms will be required. Any such analyses would require larger sample sizes than was possible here and should be carefully controlled and pre-registered to avoid “fishing” experiments.

## Self-certification statement

The authors confirm that they had no prior access to the full dataset described here.

For the Stage 1 Registered Report, the neurodevelopmental data had already been processed by DB and PT to derive the phenotypes to be used in the analysis; DFN had processed the DNA data separately to decide on the genotypes. The key tests proposed in this protocol involved putting the two strands of data together, which was deliberately not done, so that predictions could be derived without being aware of the data.

## Data availability

Phenotype data, full tables of covariances and means of all variables, and an R markdown script for generating results are available on Open Science Framework. Dataset 1: SCT genetics: Double hit hypothesis https://doi.org/10.17605/osf.io/pr54g

Exploratory work: Appendix 10, which documents additional gene-based analyses that were not part of the pre-registered plan, is also available on Open Science Framework. https://osf.io/u7dsw ( Bishop *et al*., 2019)

The data is available under a CC0 1.0 Universal license.

Ethics approval for open data is conditional on data being fully anonymised. The genotype data could be used to identify individuals and so this part of the dataset cannot be made openly available. To optimise use of the data by others, we have created and deposited synthetic data using the R package *synthpop* v1.4-3 ( Nowok *et al*., 2016), which allows other users to explore a dataset that is closely based on the original data.

## Supplementary material

Appendices 1– 9 are available on Open Science Framework ( Bishop *et al.*, 2019). Appendix 10, containing additional analyses added to version 3, are also available at this web address.
