# Supplementary material for: Stage 2 Registered Report: Variation in neurodevelopmental outcomes in children with sex chromosome trisomies: testing the double hit hypothesis
Source: Wellcome Open Res. 2021 Jun 1;3:85. Originally published 2018 Jul 19. [Version 4] doi: 10.12688/wellcomeopenres.14677.4 (PMC6134338; doi:10.12688/wellcomeopenres.14677.4)
Supplement: Peer review history

# Peer review: Reviewer response for version 4

**DOI:** 10.21956/wellcomeopenres.18694.r44186

**Published:** 2021-06-03

**Authors:** Beate St Pourcain

**Affiliations:** 1 Language and Genetics Department, Max Planck Institute for Psycholinguistics, Nijmegen, The Netherlands

I approve of the amended article.

Is the work clearly and accurately presented and does it cite the current literature?

Yes

If applicable, is the statistical analysis and its interpretation appropriate?

Yes

Are all the source data underlying the results available to ensure full reproducibility?

Yes

Is the study design appropriate and is the work technically sound?

Yes

Are the conclusions drawn adequately supported by the results?

Yes

Are sufficient details of methods and analysis provided to allow replication by others?

Yes

Reviewer Expertise:

NA

I confirm that I have read this submission and believe that I have an appropriate level of expertise to confirm that it is of an acceptable scientific standard.

# Peer review: Reviewer response for version 3

**DOI:** 10.21956/wellcomeopenres.17228.r40439

**Published:** 2021-01-04

**Authors:** David H. Skuse

**Affiliations:** 1 Behavioural Sciences Unit, UCL Great Ormond Street Institute of Child Health, London, UK

In this, the third version of their planned test of the ‘double hit’ hypothesis, Newbury *et al.* have now tested their hypothesis that the adverse impact of an additional sex chromosome on language development could be amplified by genes that are expressed from the sex chromosomes (specifically NLGN3 and NLGN4) in epistasis with specific autosomal genes NRXN1 and CNTNAP2. In my original commentary on the proposal (Version 1) I suggested that the evidence supporting the hypothesis was rather weak. However, it is undeniable that there is a considerable variation in the phenotypic presentation of the sex chromosome aneuploidies (XXX, XXY, XYY), which exceeds variation in the general population in terms of their favoured measures of language competence (see Figure 5 in Version 3). We do not yet have any satisfactory explanation for why that should be the case.

 In the current report, no association was found between phenotypic variation and specific potentially salient variants of either NRXN1 or CNTNAP2. Intriguingly, previous research that has linked a disordered language phenotype to specific minor allelic variation of CNTNAP2 was not replicated here in a typically developing twin comparison sample. This could be due to a lack of power to detect an effect, as the finding was previously replicated in many studies. Consequently, it seems reasonable to conclude the analysis that employed a novel statistical methodology (critiqued in my previous commentary), which was supposedly suitable for small sample sizes, was probably subject to a Type II error.

 The authors went on to look at alternative models of effects in a small number of candidate genes that have previously been associated with language-related phenotypes, and hence could modify the impact of any epistatic interactions between their X-linked candidates (NLGN3/4) and NRXN1 or CNTNAP2 variants. There is an image describing how these genes were selected from a SCOPUS search provided in Figure S1. In Appendix 9 an analysis is described for 23 SNPs in NRXN1 and 24 in CNTNAP2 where association was sought with language phenotypes in both the SCA and the twin samples. After Bonferroni correction, no association met statistical significance. In the supplementary material, the SNP analyses of the NRXN1 gene for variants 7-11 were omitted, presumably in error.

 Gene-based analyses were performed on the 22 genes described in Figure S1 (Appendix 5), which apparently involved ‘all known variants across the 22 candidate genes’ and their potential association with the three key language variables of interest. I am unfamiliar with this mode of analysis so cannot comment on the specific risk of false positive results. Little is said about the outcome of this analysis other than that, after Bonferroni correction, none of the p-values was ‘significant’ – meaning that (presumably) variations in these additional genes did not influence the strength of association that was the subject of the original hypothesis.

 I posited in my original commentary that CNVs could be impacting on language development in their sample of sex chromosome aneuploidies and acting as vulnerability factors. I am pleased to see that the authors are planning to undertake these analyses in the future. They may also examine the variability in the ‘escape from inactivation’ status of NLGN4X, which lies outside the PAR1 and whose inactivation is believed to be only partial.

 I also predicted that there would be least variability in the language-related phenotype of the XYY male sample and arguably the most variable phenotypes in the XXX sample, because of the relationship between NLGN4X (partially inactivated) and NLGN4Y (fully expressed). In this latest version of their paper the authors state they followed up my suggestion and provide the results of their investigation in Table 3. Table 3 as presented on p11 does not mention NLGN4 but illustrates a completely different analysis relating to NRXN1 and CNTNAP2. It is nevertheless stated that my hypothesis was not supported!

Is the work clearly and accurately presented and does it cite the current literature?

Yes

If applicable, is the statistical analysis and its interpretation appropriate?

I cannot comment. A qualified statistician is required.

Are all the source data underlying the results available to ensure full reproducibility?

Yes

Is the study design appropriate and is the work technically sound?

Yes

Are the conclusions drawn adequately supported by the results?

Yes

Are sufficient details of methods and analysis provided to allow replication by others?

Yes

Reviewer Expertise:

Neurodevelopmental disorders, sex chromosomal aneuploidies, genetic syndromes associated with intellectual disability.

I confirm that I have read this submission and believe that I have an appropriate level of expertise to confirm that it is of an acceptable scientific standard.

# Response

**Published:** 2021-05-20

**Authors:** Dorothy Bishop

**Affiliations:** University of Oxford, UK

Many thanks for these additional comments on our paper.   We have made some modifications to the main text, and in addition offer some replies here to specific points: 

 1. **Power of GSCA.** Since writing our original paper, we have done further analysis of the power of GSCA using simulations (Thompson, P. A., Bishop, D. V. M., Eising, E., Fisher, S. E., & Newbury, D. F. (2020). Generalized Structured Component Analysis in candidate gene association studies: Applications and limitations. *Wellcome Open Research*, *4*, 142. https://doi.org/10.12688/wellcomeopenres.15396.2). A type II error cannot be ruled out, but we have shown that the method works well with the kind of sample size and number of SNPs used here.  

 2, **Omission of data from table**. Thanks to the reviewer for for spotting the omission of one set of SNPs in Appendix 9. We have corrected Appendix 9 (on Open Science Framework) to now include the missing SNPs, and have updated the script used to generate the tables. 

 3. **Clarification regarding gene-based analyses**. The gene-based analyses involve a cumulative assessment of all genetic variation in a single test allowing for differences in functional variants across individuals. The results indicate that variations across the 22 genes tested do not influence language outcomes in SCT cases or twins. 

 4. **Role of CNVs.**

 Since publishing version 3, we have reported an analysis of CNVs in this sample. Once again we have null findings. See: Mountford, H. S., Bishop, D. V. M., Thompson, P. A., Simpson, N. H., & Newbury, D. F. (2020). Copy number variation burden does not predict severity of neurodevelopmental phenotype in children with a sex chromosome trisomy. *American Journal of Medical Genetics Part C: Seminars in Medical Genetics*, *184*(2), 256–266. https://doi.org/10.1002/ajmg.c.31791

 5. **Skuse predictions and Table 3.**

 Table 3 tests for putative epistatic effects of variations across *NRXN1 *and *CNTNAP2* in SCT subgroups, in line with the analyses presented elsewhere in the manuscript. These analyses do not directly test *NLGN4* but instead focus upon epistatic interactions with neuroligins. The idea is that if there is more consistent expression of *NLGN4Y*, then this might be evident as stronger impact in epistatic interactions. We wanted to credit the reviewer for the idea of expression differences in *NLGN4YX *and *NLGN4Y, *but have now reworded to clarify that the analysis in Table 3 is indirect evidence for this effect.

# Peer review: Reviewer response for version 3

**DOI:** 10.21956/wellcomeopenres.17228.r40445

**Published:** 2020-11-10

**Authors:** Beate St Pourcain

**Affiliations:** 1 Language and Genetics Department, Max Planck Institute for Psycholinguistics, Nijmegen, The Netherlands

Newbury and colleagues have extended their studies of variation in neurodevelopmental outcomes in children with sex chromosome trisomies with extensive gene-based analyses. However, none of the gene-based analyses passed the multiple testing threshold (Bonferroni correction) indicating that larger number of genes and samples will need to be screened to find evidence supporting the double-hit hypothesis.

 I have only minor comments:

1. I assume the authors mean Indels and multi-allelic variants when referring to Non-SNPs. Could the authors please re-phrase?
2. Within the exploratory analyses paragraph, it reads as if imputation was used to impute also rare variants for rare-variant analyses. “Common and rare variants were further examined using the SKAT test (MAF upper limit 0.5).” Do the authors just mean a rare variant test applied to common variants? Please clarify which variants (MAF range) were analysed with which test.
3. Could the authors please clarify what they mean with the “Zeggini” test? “A rare variant test analysis was performed using Rvtests (Zhan et al, 2016) to analyse three quantitative phenotypes: nonword repetition, language factor and global neurodevelopment index in the SCT and Twin sample sets using the Zeggini test with a MAF upper limit of 0.01.”
4. The author may consider increasing the number of digits reported for p (Appendix 9). For example, the TWA_global_p for rs2193417_G is given as 0.00 (NRXN1), which is now difficult to evaluate with respect to a multiple testing threshold of 0.0003.

Is the work clearly and accurately presented and does it cite the current literature?

Yes

If applicable, is the statistical analysis and its interpretation appropriate?

Yes

Are all the source data underlying the results available to ensure full reproducibility?

Yes

Is the study design appropriate and is the work technically sound?

Yes

Are the conclusions drawn adequately supported by the results?

Yes

Are sufficient details of methods and analysis provided to allow replication by others?

Yes

Reviewer Expertise:

NA

I confirm that I have read this submission and believe that I have an appropriate level of expertise to confirm that it is of an acceptable scientific standard.

# Response

**Published:** 2020-12-19

**Authors:** Dorothy Bishop

**Affiliations:** University of Oxford, UK

We thank the reviewer for the additional points, which allow us to add further clarification.

 1. Yes, the non-SNPs included indels and multi-alleleic SNPs.  

 2-3. Two analyses were performed within RVTESTS; the SKAT test and the Zeggini test.  

 The Zeggini test is a test of association. This is an aggregation test which computes a cumulative score of rare variant burden across the gene of interest which is then tested for association to the phenotype of interest. The test is described in doi: 10.1002/gepi.20450.  

 The Zeggini test is suited to the analysis of rare variants only, while SKAT can handle both common and rare variants within a single test. Accordingly, we applied the Zeggini test to all variants with MAF≤0.01 and the SKAT test to all variants with MAF≤0.5 (i.e. all variants across the gene, both common and rare).

 4 **.** ****Appendix 9 is not part of the additional content we added in version 3 of the manuscript - which is termed 'Exploratory analyses'. Appendix 9 was part of our original submission and was titled ‘Additional exploratory analyses’. We apologise for confusing labelling of sections.

# Peer review: Reviewer response for version 1

**DOI:** 10.21956/wellcomeopenres.15981.r33521

**Published:** 2018-09-11

**Authors:** David H. Skuse

**Affiliations:** 1 Behavioural Sciences Unit, UCL Great Ormond Street Institute of Child Health, London, UK

The Stage 2 report summarizes the findings of the proposed investigation, which aimed to test the hypothesis that neuroligin genes on the sex chromosomes (specifically NLGN4X and Y) would be expressed in excessive dosage in multiple sex chromosome trisomy (SCT) aneuploidies (XXX,XXY,XYY) and that there could be an epistatic interaction between those genes and a specific autosomal gene on Chromosome 7 (CNTNAP2), variants of which have been shown to be associated with disorders of language in particular. Accordingly, this interaction would explain the deficits in language skills characterizing SCTs, which are relatively more common in XYY than XXX individuals, with XXY males intermediate between the other two conditions.

 In the original proposal, besides the epistatic interaction with CNTNAP2, the authors also proposed an interaction between NLGN and the autosomal gene NRXN1 (chromosome 2), such that variants in that gene within putative regulatory regions could modify the impact of excessive NLGN expression.

 Essentially, the purpose of the investigation was to evaluate an intriguing ‘double-hit’ mechanism, whereby the variable impact of sex chromosome aneuploidy on language phenotypes would be explained by some sort of interaction with autosomal genetic variation, in two specific candidates (CNTNAP2 and NRXN1). The locus of the interaction was proposed to be at the synapse, reflected in variable synaptic scaffolding with functional consequences. Underlying this hypothesis was the observation that there is a transmembrane connection in the synaptic scaffolding system between CNTNAPs and Contactins/Catenins and also between Neuroligins/Neurexins as illustrated in Figure 1.

 Having reviewed the material in the original Stage 1 Proposal, I suggested there should be the most variable language phenotypes within the XXX sample, and the least variable language phenotype in the XYY sample. I expressed skepticism that the overall double-hit hypothesis would be supported by the proposed analysis, and also that the novel statistical methods to be used would truly get around the problem of limited power (which arose from the small sample size).

 The Stage 2 report (19 ^th^ July 2018) states there was wide phenotypic variation observed within the SCT groups, but no overall association could be found with the variants that had been measured on the autosomal genes CNTNAP2 or NXN1. Nor were there any specific associations within each of the trisomies investigated. The latest report reiterates much of the information about the study design that was provided by the Stage 1 report (and its April 2018 revision).

 A variety of analyses are presented for which the overall structure is represented by Figure 4. It is gratifying that measures of language impairment, for which details will be given in a future publication, did show sensitivity to the presence of sex chromosome aneuploidy status, and good correlations with the language factor. I was not surprised that the findings reported in Figure 6, which attempted to replicate previous reports on associations between specific SNPs on CNTNAP2 and neurodevelopmental impairment, showed no statistically significant result. As the authors state, replication in these complex phenotype-genotype associations is the exception rather than the rule, and there is likely to be publication bias in favor of positive results. It is commendable that the authors did not continue to look for association with alternative genes/SNPs in a fishing expedition that would have led them open to the risk of a type 1 error.

 There was no evidence found that the results varied by karyotype as I had proposed in my earlier commentary, on the grounds that there was likely to be a more variable language phenotype in XXX than XYY individuals because NLGN4X was subject to variable and partial X-inactivation (whereas NLGNY was fully expressed). Accordingly, all males with XYY would over-express NLGN4Y to the same extent, but females with XXX (in which two X-chromosomes are silenced) would express NLGN4X to a highly variable degree depending on individual differences in X-inactivation status.

 I was surprised by the comment that, in the context of a discussion about alternative models of effects, that there are many possible mechanisms of functional interaction that would not be detected by the approach taken by this study – such as functionally valid variants that lie outside the coding regions. My understanding is that many SNPs employed in this study were indeed outside coding regions (though may have had functional significance).

 There is a further comment concerning a mechanism whereby there could be modulation of expression of NLGN4X/Y by autosomal variants: a point made in my original commentary is that the regulation of NLGN4X is complex in relation to X-inactivation, and that its expression in individuals with more than one X chromosome is unpredictable for that reason in particular. This mechanism leading to variability of expression may be more pertinent than modulation by autosomal genes.

 Overall, the study was conducted with impeccable scientific rigor and exemplifies the benefits of registering a research design with Wellcome Open Research, and seeking peer review before undertaking the proposed analysis.

**Are the data able to test the authors’ proposed hypotheses by satisfying the approved outcome-neutral conditions (such as quality checks, positive controls)?**

 Yes

**Are the introduction, rationale and stated hypotheses the same as the approved Stage 1 submission? (required)**

 Yes

**Did the authors adhere precisely to the registered experimental procedures? If not, has an explanation been provided regarding any change?**

 Yes

**Are any unregistered post hoc analyses added by the authors justified, methodologically sound and informative?**

 Yes

Is the work clearly and accurately presented and does it cite the current literature?

Yes

If applicable, is the statistical analysis and its interpretation appropriate?

I cannot comment. A qualified statistician is required.

Are all the source data underlying the results available to ensure full reproducibility?

Yes

Is the study design appropriate and is the work technically sound?

Yes

Are the conclusions drawn adequately supported by the results?

Yes

Are sufficient details of methods and analysis provided to allow replication by others?

Yes

Reviewer Expertise:

Neurodevelopmental disorders, sex chromosomal aneuploidies, genetic syndromes associated with intellectual disability

I confirm that I have read this submission and believe that I have an appropriate level of expertise to confirm that it is of an acceptable scientific standard.

# Peer review: Reviewer response for version 1

**DOI:** 10.21956/wellcomeopenres.15981.r33522

**Published:** 2018-08-31

**Authors:** Beate St Pourcain

**Affiliations:** 1 Language and Genetics Department, Max Planck Institute for Psycholinguistics, Nijmegen, The Netherlands

In this Stage 2 Registered Report Newbury and colleagues studied whether the presence of an extra sex chromosome is associated with an increased rate of neurodevelopmental difficulties involving language. The authors investigate here a double-hit hypothesis, specifically that the presence of an additional sex chromosome amplifies the impact of common autosomal genetic variants residing within genes involved in synaptic functionality. The authors specifically focus on the functional network of neuroligins and study common variants within CNTNAP2 and NRXN1, and use complex analysis strategies, such as Generalised Structural Component Analysis, to overcome small sample size. Unfortunately, the sample size was small, and the authors found no support for the idea that common variants in *CNTNAP2* or *NRXN1 *are associated with the severity of language and neurodevelopmental impairments that often accompany an extra X or Y chromosome.

 However, although this study was small, the authors could make suggestions, based on their extensive expertise, how a future study should be conducted that may be able to overcome current limitations. It is highly commendable that authors followed a fully transparent analysis approach, sharing scripts and simulated comparable data. The authors may also want to highlight the transferability of their applied structural equation modelling approach.

**Are the data able to test the authors’ proposed hypotheses by satisfying the approved outcome-neutral conditions (such as quality checks, positive controls)?**

 Yes.

**Are the introduction, rationale and stated hypotheses the same as the approved Stage 1 submission? (required)**

 Yes.

**Did the authors adhere precisely to the registered experimental procedures? If not, has an explanation been provided regarding any change?**

 Yes.

**Are any unregistered post hoc analyses added by the authors justified, methodologically sound and informative?**

 Yes.

Is the work clearly and accurately presented and does it cite the current literature?

Yes

If applicable, is the statistical analysis and its interpretation appropriate?

Yes

Are all the source data underlying the results available to ensure full reproducibility?

Yes

Is the study design appropriate and is the work technically sound?

Yes

Are the conclusions drawn adequately supported by the results?

Yes

Are sufficient details of methods and analysis provided to allow replication by others?

Yes

Reviewer Expertise:

NA

I confirm that I have read this submission and believe that I have an appropriate level of expertise to confirm that it is of an acceptable scientific standard.
